# Changes in mean serum lipids among adults in Germany: results from National Health Surveys 1997-99 and 2008-11

**DOI:** 10.1186/s12889-016-2826-2

**Published:** 2016-03-08

**Authors:** Julia Truthmann, Anja Schienkiewitz, Markus A. Busch, Gert B. M. Mensink, Yong Du, Anja Bosy-Westphal, Hildtraud Knopf, Christa Scheidt-Nave

**Affiliations:** Department of Epidemiology and Health Monitoring, Robert Koch Institute, Berlin, Germany; Institute of Nutritional Medicine, University of Hohenheim, Stuttgart, Germany

**Keywords:** Total cholesterol, Triglycerides, High density lipoprotein-cholesterol, Lifestyle, Lipid-lowering medication

## Abstract

**Background:**

Monitoring of serum lipid concentrations at the population level is an important public health tool to describe progress in cardiovascular disease risk control and prevention. Using data from two nationally representative health surveys of adults 18–79 years, this study identified changes in mean serum total cholesterol (TC), high-density lipoprotein cholesterol (HDL-C), and triglycerides (TG) in relation to changes in potential determinants of serum lipids between 1997–99 and 2008–11 in Germany.

**Methods:**

Sex-specific multivariable linear regression analyses were performed with serum lipids as dependent variables and survey wave as independent variable and adjusted for the following covariables: age, fasting duration, educational status, lifestyle, and use of medication.

**Results:**

Mean TC declined between the two survey periods by 13 % (5.97 mmol/l vs. 5.19 mmol/l) among men and by 12 % (6.03 mmol/l vs. 5.30 mmol/l) among women. Geometric mean TG decreased by 14 % (1.66 mmol/l vs. 1.42 mmol/l) among men and by 8 % (1.20 mmol/l vs. 1.10 mmol/l) among women. Mean HDL-C remained unchanged among men (1.29 mmol/l vs. 1.27 mmol/l), but decreased by 5 % among women (1.66 mmol/l vs. 1.58 mmol/l). Sports activity and coffee consumption increased, while smoking and high alcohol consumption decreased only in men. Processed food consumption increased and wholegrain bread consumption decreased in both sexes, and obesity increased among men. The use of lipid-lowering medication, in particular statins nearly doubled over time in both sexes. Among women, hormonal contraceptive use increased and postmenopausal hormone therapy halved over time. The changes in lipid levels between surveys remained significant after adjusting for covariables.

**Conclusion:**

Serum TC and TG considerably declined over one decade in Germany, which can be partly explained by increased use of lipid-lowering medication and improved lifestyle among men. The decline in serum lipids among women, however, remains unexplained.

**Electronic supplementary material:**

The online version of this article (doi:10.1186/s12889-016-2826-2) contains supplementary material, which is available to authorized users.

## Background

Increased low density lipoprotein-cholesterol (LDL-C) and total cholesterol (TC) serum concentrations are among the major modifiable risk factors of cardiovascular disease (CVD) amenable to life style and pharmaceutical intervention [1]. Other dyslipidaemias, including low high density lipoprotein cholesterol (HDL-C) and elevated triglyceride (TG) levels are markers of increased CVD risk; however, the evidence for a causal relationship and practical implications for treatment are less clear [2, 3]. Monitoring of serum lipid concentrations and other major CVD risk factors at the population level is an important public health tool to describe progress in CVD risk control and prevention. Analyses conducted by the Global Burden of Metabolic Risk Factors of Chronic Diseases Collaborating Group showed that mean serum cholesterol declined by 0.2 mmol/l per decade for men and women in high-income regions between 1990 and 2008 [4]. Serum lipid levels are determined by genetic [5] as well as acquired factors, such as lifestyle [6], in particular diet, physical activity, and smoking, use of lipid-lowering medications, use of other medications e.g. hormonal contraceptives [7] and postmenopausal hormone therapy [8], and diseases [9]. Trends in serum lipids and their underlying determinants are likely to differ between populations due to country-specific trends in lifestyle and medication use; hence country-specific data are needed for public health recommendations and health policy consulting. Significant declines in serum lipids at the population level have been reported from the United States (US) [10] and several European countries [11–13]. Previous analyses in Germany were limited to the population of Western Germany and the time period between 1984 and 1998 [14] and need an update. Based on data from two nationally representative health interview and examination surveys conducted in 1997–99 and in 2008–11, we examined changes in serum TC, TG, and HDL-C among 18–79-year old adults in Germany between the two survey periods in relation to changes in lifestyle and use of lipid-lowering medication.

## Methods

### Study design

Data from the German National Health Interview and Examination Survey 1997–99 (GNHIES98) and the subsequent German Health Interview and Examination Survey for Adults 2008–11 (DEGS1) were used. The design and study protocol of both surveys has been previously described in detail [15, 16]. In brief, in both surveys participants were selected using a two-stage clustered sampling procedure. Within the community, which was the primary sampling unit, individuals are sampled randomly from local population registries. GNHIES98 was approved by the Board of the Federal Commissioner for Data Protection Berlin and DEGS1 was approved by the Federal and State Commissioners for Data Protection and the Charité - Universitätsmedizin Berlin ethics committee (No. EA2/047/08). The implementation of the study conforms to the principles of the Helsinki Declaration. Informed consent was obtained from all participants prior to inclusion in the study. GNHIES98 comprises 7124 participants and DEGS1 comprises 7115 participants at the age of 18–79 years, who had participated in the examination part.

### Laboratory analyses

In both surveys, venous blood samples were taken at the study centres. For logistic reasons and in order to enhance participation, appointments were scheduled throughout the day at the participants’ convenience. The time of the blood sampling as well as the number of hours since last meal were recorded. Fasting duration was higher in the 2008–11 survey compared to the 1997–99 survey due to a change in the instructions provided in the invitation letter. In the 1997–99 survey blood was drawn in a non-fasting state. In the 2008–11 survey, participants, except those with diagnosed diabetes, were encouraged to schedule morning appointments after an overnight fasting period of at least eight hours; for appointments later during the day, a fasting period of at least four hours was recommended. Blood samples were processed within one hour and stored at −40 °C until analysis in the central laboratory unit at the Robert Koch Institute. Serum lipid levels were determined by an enzymatic procedure, based on the CHOD-PAP method (TC, HDL-C) and the GPO-PAP method (TG). While the principle of measurement remained the same, the analytic system for serum lipids changed during the study period (GNHIES98: MEGA, Merck, Germany; DEGS1: Architect ci2800, Abbott, Germany). All processes of laboratory analyses were performed in accordance with standard operating procedures.

### Covariables

Information on lifestyle was assessed using a self-administered questionnaire. Smoking was assessed using five categories in GNHIES98 (“yes, daily”, “yes, occasionally”, “ex-smoker, less than 12 month”, “ex-smoker, more than 1 year”, “never”) and four categories in DEGS1 (“yes, daily”, “yes, occasionally”, “ex-smoker”, “never”). For the present analyses, current smoking was categorized as “no”, “occasionally”, and “daily”. Sports activity was assessed by five categories (“no”, “less than 1 h”, “regularly 1–2 h”, ”regularly 2-4 h”, “regularly more than 4 h”) in both surveys and re-categorised to “no”, “up to 2 h per week”, and “regularly, at least 2 h per week”.

In GNHIES98, a self-administered questionnaire included questions on the frequency of food and non-alcoholic beverage consumption during the last 12 months. In addition the usual amounts of alcoholic drinks per occasion were asked. In DEGS1 a semi-quantitative self-administered food frequency questionnaire was used to assess frequency and portion size of food and beverage consumption (during the last 4 weeks). Dietary components that influence serum lipid levels are mainly saturated fatty acids, polyunsaturated fatty acids, trans fatty acids (TFA), cholesterol and fibre [17]. Due to limited information on food intake, the ratio of polyunsaturated to saturated fatty acids or even changes in white to red meat consumption could not be taken into account. Based on the data available, we included consumption of wholegrain bread [18], processed foods [19], coffee [20] and alcohol [21] in our models, which were significantly associated with serum lipid levels in previous studies. Detailed information on the categorization of dietary variables is shown in Additional file 1: Table S1. High alcohol consumption was defined as more than 20 g per day for men and more than 10 g for women [22].

Anthropometric measurements were performed in light clothing without shoes in GNHIES98 and in underwear without shoes in DEGS1. Body height was measured with a precision of 0.1 cm and body weight was measured with a precision of 0.1 kg. Body mass index (BMI) was calculated as body weight (kg) divided by height squared (m^2^) and categorized as normal weight (BMI < 25 kg/m^2^), overweight (25 kg/m^2^ ≤ BMI < 30 kg/m^2^), and obese (BMI > 30 kg/m^2^) according to WHO recommendations [23].

Any medication taken within seven days prior to the interview was recorded and validated based on the original containers brought to the study centre [24]. Classification was based on the most currently available version of the Anatomical Therapeutic Chemical (ATC) classification system. We considered the following medications known to affect blood lipids: lipid-lowering medications (C10) especially statins (C10AA, C10BA), hormonal contraceptives (G02B, G03A, G03HB01, G03FB03), and postmenopausal hormone therapy (G03C, G03D, G03F excluding G03FB03, G03HB).

The educational status was assessed using a self-administered questionnaire and categorized as “low”, “middle”, and “high” according to Comparative Analysis of Social Mobility in Industrial Nations (CASMIN) classification system [25].

### Analysis

Participants with missing values on serum lipid levels, lifestyle and use of medication were excluded, resulting in a final study sample of 6432 subjects in GNHIES98 (3144 men, 3288 women) and 6604 subjects in DEGS1 (3146 men, 3458 women). All analyses were stratified for sex. Survey-specific means, percentages and 95 % confidence intervals were calculated. Differences between survey-specific means of serum lipids were estimated with the t-test and differences between categories of lifestyle and medication use with the Rao-Scott Chi-Square test. The distribution of TG was skewed, therefore geometric means were calculated. Kernel density distributions of TC, HDL-C, and TG were plotted. Linear regression analyses were performed with TC, HDL-C, and TG (natural log-transformed) as dependent variables and survey wave (GNHIES98, DEGS1) as independent variable (Model 1). Model 2 was additionally adjusted for age. As TC might be non-linearly associated with age, with highest values at around 50 to 60 years [4], age was included as a cubic spline with knots set at the 20^th^, 40^th^, 60^th^, and 80^th^ percentile. Model 2 for TG was additionally adjusted for fasting duration (“less than four hours”, “four to eight hours”, “more than eight hours”). Model 3 additionally included the following covariables: educational status, smoking, coffee consumption, processed foods consumption, wholegrain bread consumption, high alcohol consumption, sports activity, BMI category, lipid-lowering medication, and among women hormonal contraceptive use and postmenopausal hormone therapy use. Correlation coefficients between lifestyle variables were all lower than 0.2 and variance inflation factors of all explanatory variables were lower than 2.5. To improve the interpretability of the results from regression analyses based on natural log-transformed TG values, we calculated the antilog of the regression coefficient.

All statistical analyses were performed using survey procedures for complex samples in SAS 9.4 (SAS Institute, Cary, NC). Analyses were weighted using a weighting factor to correct deviations from the population structure in Germany with regard to age, sex, educational status, federal state, and type of municipality as of 31.12.1997 for GNHIES98 and 31.12.2010 for DEGS1 [26]. For the trend analyses the GNHIES98 data were additionally age-standardized for the population on 31 December 2010. P values less than 0.05 were defined as statistically significant based on two-sided tests.

In sensitivity analyses missing values were replaced by the Fully Conditional Specification method using five sets of imputation. For this purpose all analysis variables and further auxiliary variables (weighting factor, sample point, systolic blood pressure, and waist circumference) were used. Data were imputed separately for both sexes and for both study periods. Combined parameter estimates, confidence intervals and P values were reported and ranges of the model fit (min/max) were presented.

## Results

Results for most study variables significantly changed between the two survey periods (Tables 1 and 2). Compared to 1997–99 men and women were significantly better educated in 2008–11. In both sexes, coffee consumption, processed foods consumption and sports activity increased, while wholegrain bread consumption decreased. The prevalence of current smokers and persons with high alcohol consumption significantly decreased among men, but not among women. In contrast, obesity significantly increased among men, but not among women. The prevalence of persons using lipid-lowering medication more than doubled between the two surveys in both sexes. Among women, the prevalence of hormonal contraceptive users significantly increased between survey periods, while the prevalence of postmenopausal hormone therapy users more than halved, between 1997–99 and 2008–11.

**Table 1:** Study characteristics in 1997–99 and 2008–11 among men

		GNHIES98^a^ *N* = 3144	DEGS1^b^ *N* = 3146	*P* ^b^
Age (mean, years)		44.3 (43.5–45.1)	46.5 (45.8–47.2)	.600
Fasting duration (%)	<4 h	28.5 (26.3–30.9)	10.6 (9.3–12.0)	**<.001**
	4 – 8 h	48.9 (46.8–51.0)	38.3 (36.3–40.5)	
	>8 h	22.6 (20.6–24.6)	51.1 (49.0–53.1)	
Educational status (%)	Low	48.2 (45.1–51.3)	35.0 (32.2–37.9)	**<.001**
	Middle	36.7 (34.3–39.2)	47.8 (45.3–50.3)	
	High	15.1 (13.4–17.0)	17.2 (15.4–19.2)	
Lifestyle				
Current smoking (%)	No	61.8 (59.5–64.1)	67.7 (65.3–70.1)	**.009**
	Occasionally	6.0 (5.1–7.0)	6.4 (5.4–7.7)	
	Daily	32.2 (29.9–34.6)	25.8 (23.6–28.2)	
Daily coffee consumption (%)		70.5 (68.3–72.6)	77.3 (75.3–79.2)	**<.001**
Daily processed foods consumption (%)		65.4 (63.3–67.4)	68.6 (66.3–70.8)	**<.001**
Wholegrain bread consumption (%)	Never	13.8 (12.0–15.7)	16.7 (15.0–18.5)	**<.001**
	Less than daily	51.8 (49.8–53.9)	58.1 (55.9–60.3)	
	Daily	34.4 (32.0–36.7)	25.2 (23.1–27.3)	
High alcohol consumption (%)		26.1 (24.0–28.4)	18.6 (16.9–20.3)	**<.001**
Sports activity (%)	No	44.9 (42.6–47.3)	32.2 (30.1–34.5)	**<.001**
	Up to 2 h per week	31.8 (30.1–33.6)	38.4 (36.1–40.8)	
	Regularly, at least 2 h per week	23.3 (21.4–25.2)	29.3 (27.4–31.4)	
BMI category (%)	Normal weight	32.1 (29.8–34.5)	32.7 (30.6–35.0)	**<.001**
	Overweight	48.6 (46.6–50.6)	43.7 (41.6–45.9)	
	Obese	19.3 (17.7–21.0)	23.5 (21.3–25.9)	
Use of Medication				
Lipid-lowering medication (%)		5.8 (4.9–7.0)	10.9 (9.7–12.3)	**<.001**
Statins (%)		3.5 (2.8–4.3)	9.8 (8.5–11.1)	**<.001**
Serum Lipids				
Total cholesterol (mean, mmol/l)		5.97 (5.91–6.03)	5.19 (5.13–5.24)	**<.001**
Triglycerides (geometric mean, mmol/l)		1.66 (1.61–1.71)	1.42 (1.38–1.45)	**<.001**
High density lipoprotein (mean, mmol/l)		1.29 (1.27–1.31)	1.27 (1.25–1.29)	.170

Differences between categorical variables were estimated with the Rao-Scott Chi-Square test and differences between mean serum lipid levels were estimated with the t-test. *P* values < 0.05 were considered statistical significant (bold). Mean estimates are weighted: ^a^Standardized to population structure as of 31^st^ December 1997; ^b^Standardized to population structure as of 31st December 2010

Number of missing values (GNHIES98/DEGS1): Fasting duration (120/23), educational status (87/24), current smoking (66/20), daily coffee consumption (81/69), daily processed food consumption (78/59), wholegrain bread consumption (101/78), high alcohol consumption (79/61), sports activity (81/106), BMI category (15/20), lipid-lowering medication/statins (12/11), total cholesterol (172/38), triglycerides (172/34), high density lipoprotein (174/34)

**Table 2:** Study characteristics in 1997–99 and 2008–11 among women

		GNHIES98^a^ *N* = 3288	DEGS1^b^ *N* = 3458	*P* ^b^
Age (mean, years)		46.2 (45.4–46.9)	47.5 (46.8–48.2)	.870
Fasting duration (%)	<4 h	27.6 (25.6–29.8)	8.5 (7.4–9.8)	**<.001**
	4–8 h	42.1 (40.0–44.2)	46.3 (44.3–48.4)	
	>8 h	30.3 (28.3–32.3)	45.2 (43.1–47.2)	
Educational status (%)	Low	50.5 (47.4–53.6)	36.2 (33.7–38.7)	**<.001**
	Middle	40.6 (38.2–43.0)	51.3 (48.9–53.6)	
	High	8.9 (7.6–10.5)	12.6 (10.8–14.6)	
Lifestyle				
Current smoking (%)	No	70.9 (69.0–72.7)	72.8 (70.7–74.7)	.790
	Occasionally	6.0 (5.1–7.0)	5.8 (4.8–6.9)	
	Daily	23.2 (21.3–25.1)	21.5 (19.8–23.3)	
Daily coffee consumption (%)		71.3 (69.1–73.5)	78.2 (76.4–79.9)	**<.001**
Daily processed foods consumption (%)		52.4 (50.0–54.8)	62.3 (60.1–64.5)	**<.001**
Wholegrain bread consumption (%)	Never	7.3 (6.2–8.6)	8.9 (7.7–10.3)	**<.001**
	Less than daily	45.9 (43.4–48.5)	51.9 (49.4–54.4)	
	Daily	46.8 (44.3–49.2)	39.2 (36.7–41.8)	
High alcohol consumption (%)		11.7 (10.2–13.5)	13.3 (12.0–14.7)	.300
Sports activity (%)	No	50.7 (48.1–53.3)	32.9 (30.8–35.1)	**<.001**
	Up to 2 h per week	34.0 (31.8–36.2)	45.2 (43.0–47.4)	
	Regularly, at least 2 h per week	15.4 (13.9–17.0)	21.9 (20.2–23.7)	
BMI category (%)	Normal weight	46.5 (44.2–48.9)	47.5 (45.3–49.7)	.160
	Overweight	30.6 (28.9–32.5)	29.2 (27.3–31.1)	
	Obese	23.2 (21.3–25.1)	23.4 (21.4–25.4)	
Use of Medication				
Lipid-lowering medication (%)		5.4 (4.5–6.4)	8.6 (7.5–9.8)	**<.001**
Statins (%)		2.9 (2.2–3.7)	7.7 (6.7–8.7)	**<.001**
Hormonal contraceptives (%)		16.8 (15.3–18.4)	21.2 (19.5–23.0)	**<.001**
Postmenopausal hormone therapy (%)		11.7 (10.4–13.2)	5.0 (4.2–6.0)	**<.001**
Serum Lipids				
Total cholesterol (mean, mmol/l)		6.03 (5.98–6.08)	5.30 (5.24–5.37)	**<.001**
Triglycerides (geometric mean, mmol/l)		1.20 (1.17–1.23)	1.10 (1.07–1.27)	**<.001**
High density lipoprotein (mean, mmol/l)		1.66 (1.64–1.69)	1.58 (1.56–1.60)	**<.001**

Differences between categorical variables were estimated with the Rao-Scott Chi-Square test and differences between mean serum lipid levels were estimated with the t-test. *P* values < 0.05 were considered statistical significant (bold). Mean estimates are weighted: ^a^Standardized to population structure as of 31^st^ December 1997; ^b^Standardized to population structure as of 31st December 2010

Number of missing values (GNHIES98/DEGS1): Fasting duration (151/9), educational status (107/24), current smoking (95/22), daily coffee consumption (106/55), daily processed food consumption (99/47), wholegrain bread consumption (114/63), high alcohol consumption (102/48), sports activity (114/92), BMI category (37/30), lipid-lowering medication/statins (13/13), hormonal contraceptives (13/13), postmenopausal hormone therapy (13/13), total cholesterol (196/40), triglycerides (196/36), high density lipoprotein (196/37)

**Fig. 1:**
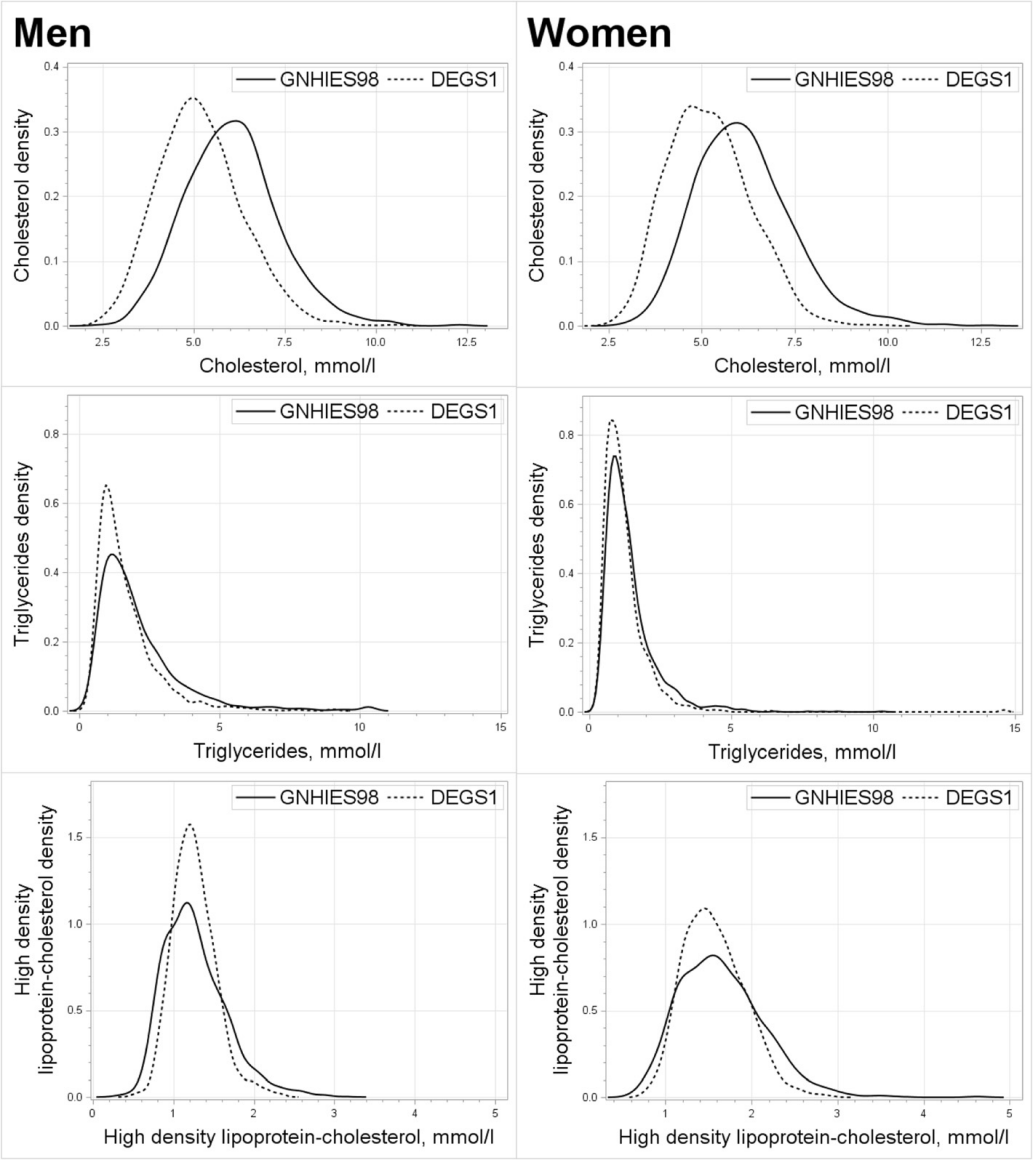
Change in serum lipid levels among men and women in 1997–99 and 2008–11. All figures are weighted population estimates: Standardized to population structure as of 31^st^ December 2010

From 1997–99 to 2008–11 mean serum TC decreased by 13 % among men and by 12 % among women (Tables 1 and 2). Geometric mean TG decreased by 14 % in men and by 8 % in women. Mean HDL-C decreased by 5 % among women only. In both sexes TC distributions were shifted to the left in 2008–11 compared to 1997–99 (Fig. 1). Distributions of HDL-C and TG did not shift, but distributions were skewed and showed higher variability in 1997–99 compared to 2008–11.

In linear regression models (Table 3) the age-adjusted decline in mean TC was 0.83 mmol/l among men and 0.78 mmol/l among women. The age-adjusted decline in mean HDL-C was 0.08 mmol/l in women. After adjusting for age and fasting period the mean of TG declined by 8 % in men and by 5 % in women. The decline in TC between the surveys was only partly explained when adjusting for age and additional covariables among men (Model 3). Similarly, the decline in TG among men between survey periods was reduced, even after adjustment for age, fasting duration and additional covariables. The decline in TC, TG, and HDL-C among women persisted with adjustment for age and additional covariables. In general, the results of the complete case analysis are comparable to the multiple imputation analysis (Additional file 2: Table S2), but a smaller part of the difference between both survey periods among men was explained using multiple imputed data.

**Table 3:** Linear regression models for serum lipids with survey wave (DEGS1 vs. GNHIES98) as independent variable

	Men					Women				
	Beta	95 % Confidence interval		*P*	R^2^	Beta	95 % Confidence interval		*P*	R^2^
		Lower	Upper				Lower	Upper		
Total cholesterol (mmol/l)										
Model 1	−0.845	−0.925	−0.765	**<.001**	.1087	−0.781	−0.861	−0.701	**<.001**	.0975
Model 2^a^	−0.828	−0.904	−0.753	**<.001**	.2559	−0.782	−0.855	−0.708	**<.001**	.2977
Model 3^b^	−0.779	−0.856	−0.702	**<.001**	.2833	−0.788	−0.865	−0.710	**<.001**	.3147
Triglycerides (log-transformed; mmol/l)										
Model 1	−0.177	−0.210	−0.143	**<.001**	.0225	−0.098	−0.133	−0.062	**<.001**	.0090
Model 2^a,c^	−0.088	−0.122	−0.054	**<.001**	.1298	−0.050	−0.084	−0.017	**.004**	.1584
Model 3^b,c^	−0.081	−0.115	−0.047	**<.001**	.2315	−0.050	−0.084	−0.017	**.003**	.2817
High density lipoprotein cholesterol (mmol/l)										
Model 1	−0.016	−0.040	0.007	.170	.0006	−0.081	−0.112	−0.051	**<.001**	.0094
Model 2^a^	−0.016	−0.039	0.007	.170	.0089	−0.081	−0.111	−0.051	**<.001**	.0168
Model 3^b^	−0.007	−0.031	0.017	.560	.1330	−0.099	−0.127	−0.071	**<.001**	.1571

Differences between mean serum lipid levels were estimated with the t test. *P* values < 0.05 were considered statistical significant (bold). All models are weighted population estimates: standardized to population structure as of 31^st^ December 2010

^a^Adjusted for age

^b^Adjusted for age, educational status, current smoking, coffee consumption, processed food consumption, wholegrain bread consumption, high alcohol consumption, sports activity, body mass index category, use of lipid-lowering medication, hormonal contraceptives, and postmenopausal hormone therapy

^c^Additionally adjusted for fasting duration

The associations of TC, TG, and HDL with covariables included in multiple linear regression models are presented in Additional file 3. Normal weight, non-smoking, and no high alcohol consumption were independently related to lower TC among men, but not among women (Additional file 3: Table S4). In both sexes, lower TC was significantly and independently associated with the use of lipid-lowering medication. Among women, lower TC was significantly and independently associated with postmenopausal hormone therapy use, while hormonal contraceptive use showed the opposite association. Lower TG concentrations were independently related to non-smoking, increased sports activity, normal weight, and daily processed foods consumption in both sexes (Additional file 3: Table S5). An independent and inverse relationship between coffee consumption and serum TG was more pronounced among men while higher intake of wholegrain bread was significantly related to lower TG in men only. The use of lipid-lowering medication was related to higher serum TG in men and women, as was hormonal contraceptive use in women. Non-smoking, daily coffee consumption, high alcohol consumption and normal weight were independent determinants of higher HDL-C in both sexes (Additional file 3: Table S6). An independent and positive relationship between sports activity and HDL-C was more pronounced in men, while higher wholegrain bread intake and higher HDL-C correlated among men only. Less than daily processed foods consumption was associated with higher HDL-C values in women only. Lipid-lowering medication use significantly correlated with lower HDL-C among men. Among women, hormonal contraceptive use was significantly related to higher HDL-C.

## Discussion

Between both German National Health Survey periods 1997–99 and 2008–11 TC (−13 and −12 %) and TG levels (−14 and −8 %) decreased in men and women, whereas HDL-C decreased in women only (−5 %). However, after adjustment for age and fasting duration almost half of the decline in mean TG was explained among men and women. Changes in lifestyle and use of medication partly explained decreasing TC and TG among men, but the survey effect remained significant even after consideration of these covariables. Decreasing TC and HDL-C levels among women remain unexplained.

The decline in serum TC observed in the present study for Germany is comparable to findings from northern Sweden, where mean TC levels decreased from 6.38 mmol/l to 5.78 mmol/l (−9 %) in men and from 6.32 mmol/l to 5.51 mmol/l (−13 %) in women from 1986 to 2004 [11]. Similarly, TC levels decreased from 5.8 mmol/l to 5.3 mmol/l (−9 %) in men and from 5.6 mmol/l to 5.1 mmol/l (−9 %) among women in Finland from 1992 to 2007 [13], with a 20 % decline observed from 1982 to 2007. In the US the decline in TC was lower. Between 1999–2002 and 2007–10 TC levels declined from 5.23 mmol/l to 5.02 mmol/l (−4 %) among men and from 5.28 mmol/l to 5.13 mmol/l (−3 %) among women. Over the same time period, HDL-C increased from 1.19 mmol/l to 1.22 mmol/l (3 %) among men and from 1.46 mmol/l to 1.49 mmol/l among women (2 %), and serum TG levels decreased from 1.49 mmol/l to 1.34 mmol/l (−10 %) among men and from 1.30 mmol/l to 1.15 mmol/l (−12 %) among women [10].

We found that the prevalence of persons using lipid-lowering medication nearly doubled between 1997–99 and 2008–11 from 6 to 11 % among men and from 4 to 10 % among women. Not surprisingly, mainly statin use increased. Statin prescription in Germany [27] as in other European countries [28] dramatically increased, ever since statins were recommended as first-line medication for patients with high low-density lipoprotein cholesterol [29]. Comparable to our results the use of lipid-lowering medication in north Sweden increased between 1994 and 2004 from almost 2 to 10 % among men and women [11]. In Finland, lipid-lowering medication use increased from 4 % in 1992 to 18 % in 2007, as compared to 2 % in 1992 and 8 % in 2007 among women [13]. The prevalence of US adults taking lipid-lowering medication started from higher baseline levels with increases from 9 % in 1999–2002 to 16 % in 2007–2010 [10]. In these previous studies increased use of lipid-lowering medications explained about 0.1 mmol/l of the decline in TC levels [11, 13, 30]. That leads us to assume that the change in medication use had a similar impact in Germany.

We found an inverse association between use of lipid-lowering medication and TG levels. Similarly, between 1996–97 and 2006–07 increasing TG levels were found among French adults [12]. However, a previous review on placebo-controlled randomized controlled trials and uncontrolled before-and-after trials reported an inverse association between the dose of atorvastatin and TG levels [31]. Our unexpected finding might be due to lifestyle differences between the group of users and non-users of lipid-lowering medication. For instance, increased TG levels are associated with changes in dietary habits when saturated fats are replaced by carbohydrates [32].

In the present study, a number of lifestyle-related factors, known to affect serum lipids, significantly changed over time and might have contributed to explain the observed changes in serum lipids. Most consistently, sports activity and coffee consumption increased over time. As previously described [33, 34], we observed a significant association between higher sports activity and lower serum TG as well as higher HDL-C. However, these associations were more pronounced among men than women in the present study. Thus, improved sports activity may have contributed to decreases in TG. We found a positive association of daily coffee consumption with HDL-C and an inverse association with TG. However, we had no information on the coffee brewing method, which may have some impact on the strength of the association [20]. In contrast to our findings, a previous meta-analysis of randomized controlled trials [20] showed increasing TC and TG values among coffee drinkers, but no association with HDL-C.

In the present study, significant reductions in tobacco use and alcohol consumption as well as increases in obesity over time were observed among men, while no significant changes were evident among women. Daily smoking decreased from 32 % in 1997–99 to 26 % in 2008–11 among men. This change most likely reflects governmental actions, especially the tobacco tax increases from 2002 to 2005, and the ban on smoking in public places since 2007 [35]. However, in northern Sweden where smoking prevalence decreased between 1994 and 2004 by 7 % (from 18 to 11 %) among men and by 8 % (from 27 to 19 %) among women, the impact of smoking on the population TC level was rather low [11]. As expected based on previous evidence, we observed a consistent and positive relationship between alcohol consumption and HDL-C in both sexes [21].

Dietary fat quality and consumption of dietary fibre have an impact on serum lipid levels [17], but due to limited availability of food intake data we could only consider consumption of wholegrain bread and consumption of processed foods in our models. However, these dietary variables did not consistently show the expected associations with serum lipids. Besides, the frequency of wholegrain bread consumption decreased and frequency of processed foods consumption increased during the study period. In Finland changes in dietary fat and cholesterol intake explained 0.7 mmol/l of the serum cholesterol decline from 1982 to 2007 [13]. Due to national prevention programmes which aimed to decrease CVD risk factors consumption of butter decreased, while consumption of low-fat milk products, vegetables, fruits, and berries increased [13]. Also in northern Sweden the trend in TC is mainly due to favourable changes in dietary habits from 1986 to 1999 [36]. In contrast, in the US no significant changes in diet could be observed regarding intake of total fat, saturated fat, polyunsaturated fat and dietary cholesterol from 1988–94 to 2007 [30]. The results of the German Nutrition Survey 1998 [37] and the National Consumption study 2005–06 [38] suggest no considerable change in total fat intake among men and women. Furthermore, a regional study among children found no substantial changes in fat quantity and quality from 2000 to 2010 [39]. As previously reported, changes in dietary habits among adults in Germany between 1997–99 to 2008–11 are characterized by increased fruit consumption as well a decrease in the frequency of raw and cooked vegetables consumption [40].

TFA seem to increase CVD risk and, among others, increase serum TC levels and decrease HDL-C levels [41]. The main sources of TFA are commercially hydrogenated oils, dairy fats and meats [42]. Due to societal pressure food producers reduced TFA content in processed foods during the last decade [42, 43]. The estimated mean intake of TFA in Germany was 1.6 gram per day (0.7 % energy) in 2013 [44]. Other studies suggested, that the changes explain about 0.04 mmol/l [45] to 0.08 mmol/l [13] of the TC decline. The decreasing TFA content could not be considered from our data.

Hormonal contraceptive use, known to increase TC, TG, and HDL-C [7], increased over time. Postmenopausal hormone therapy use, known to decrease TC and to increase HDL-C [8], substantially decreased in Germany between the two survey periods as in many other countries following the publication of the Women’s Health Initiative [46]. The small decline of HDL-C among women was unexpected, but a similar decline of HDL-C was also found between 1990 to 1994 among both sexes in northern Sweden (MONICA project), while HDL-C increased considering the period between 1986 and 1994 [47]. Among women HDL-C values decreased from 1.56 mmol/l in 1990 to 1.51 mmol/l in 1994 on a similar level as observed in this study. HDL-C increased among Non-Hispanic white adults in the US between 1999–2002 and 2007–10 [10], but comparisons are difficult, since HDL-C is known to be considerably lower in the US compared to the German adult population even after adjustment for differences in analytic methods and lifestyle variables including alcohol consumption [48].

The major strength of the present study is the population-based design which permits assessment of changes in TC, HDL-C and TG among adults in Germany over time at the national level. The observed results are generalizable to the German resident adult population, because analyses were based on large nationwide samples of the resident population and survey weights were applied accounting for the complex sampling design and non-response. There are a number of limitations. First, the model fit of the presented models is rather low. This is not surprising since this population wide sample is supposed to have a high biologic and life style variability and important determinants of lipid measures were not considered. Genetic factors could not be considered, but would also not be expected to contribute essentially to explain changes over time which was the major objective of the present analysis. Available information on food intake as well as changes in food composition over the years (like lowering of fat content in meat and changes in TFA in processed foods) was incomplete, and more refined dietary data may have contributed to the model fit and explain the change in mean serum lipids over time probably much better. Consideration of additional information that was available and comparable between the two survey periods (e. g. diagnosed diabetes mellitus, hypertension, glycated haemoglobin A1c) did not materially improve the models and did not change the interpretation of the results (Additional file 2: Table S3). Secondly, LDL-C was measured directly only in the more recent survey (DEGS1, 2008–11). Calculation of LDL-C using the Friedewald equation would have been possible, but not valid at serum triglyceride concentrations ≥4.5 mmol/l. We hence believe that inclusion of calculated LDL-C would not strengthen our analysis which focused on changes in lipid measurement distribution over time. Third, changes in the analytic system, albeit not in the serum lipid measurement method occurred between the two surveys. Calibration based on parallel measurement was not feasible, since the measurement device from GNHIES98 (MEGA, Merck, Germany) was no longer available. However, previous studies have shown that even results obtained with different measurement principles are highly correlated with differences in the range of about 0.1 mmol/l [49]. Finally, guidelines recommend measuring TG at a fasting state [1, 29]. However, blood sampling in both German national health surveys could not be generally conducted in a fasting state, as survey visits had to be offered over the entire day. Hours since last food intake were recorded in both surveys and thus we were able to adjust linear regression models of TG for fasting duration.

## Conclusions

Mean serum TC and TG declined considerably among adults in Germany between the 1997–99 and 2008–11 national health interview and examination surveys. The decline over time was more pronounced among men than among women, and was partly explained by increased use of lipid-lowering medication and improved lifestyle among men only. HDL-C persisted in men and slightly declined among women, which was not explained by changes in lifestyle or decrease in postmenopausal hormone therapy. The influence of diet on the trend of serum lipid levels could not be sufficiently taken into account in this study. A small impact of the change of the analytic system cannot be ruled out. Changes over time may also differ by subgroups of the population which needs further investigation.

## Additional files

Additional file 1: Table S1. Dietary Variables in GNHIES98 and DEGS1 (PDF 8 kb) Additional file 2: Table S2. Linear regression models for serum lipids with survey wave (DEGS1 vs. GNHIES98) as independent variable based on multiple imputed data. **Table S3.** Linear regression models for serum lipids with survey wave (DEGS1 vs. GNHIES98) as independent variable and additional explanatory variables. (DOCX 29 kb) Additional file 3: Table S4. Linear regression models for total cholesterol. **Table S5.** Linear regression models for triglycerides. **Table S6.** Linear regression models for high density lipoprotein. (DOCX 47 kb)

## Abbreviations

CVD: cardiovascular disease
HDL-C: high density lipoprotein-cholesterol
LDL-C: low density lipoprotein-cholesterol
TC: total cholesterol
TG: triglycerides
US: United States
