# Supplementary material for: Changes in mean serum lipids among adults in Germany: results from National Health Surveys 1997-99 and 2008-11
Source: BMC Public Health. 2016 Mar 8;16:240. doi: 10.1186/s12889-016-2826-2 (PMC4784325; doi:10.1186/s12889-016-2826-2)
Supplement: Additional file 1: Table S1. — Dietary Variables in GNHIES98 and DEGS1 (PDF 8 kb) [file 12889_2016_2826_MOESM1_ESM.pdf]

Changes in mean serum lipids among adults in Germany: Results from National Health Surveys 1997-99 and 2008-11
Additional File 1

Tab. S1 Dietary Variables in GNHIES98 and DEGS1

Dietary variable	GNHIES98	DEGS1	Categories
Daily coffee consumption	One item:<br>Coffee	One item:<br>Coffee	Yes<br>No
Daily processed foods consumption	Six items:<br>Fast food, fried potatoes, cakes and cookies, crisps and cracker, candy and chocolate, soft drinks	Twelve items:<br>Hamburger/kebab, fried sausages, pizza, fried potatoes, French fries, cakes, cookies, crisps, cracker, candy, chocolate, soft drinks	Yes<br>No
Wholegrain bread consumption	One item:<br>Wholegrain bread	One item:<br>Wholegrain bread	Never<br>Less than daily<br>Daily
High alcohol consumption	Four items:<br>Non-alcoholic-beer, beer, wine, spirits	Five items:<br>Non-alcoholic-beer, beer, wine, spirits, cocktails	Yes<br>No
