# Supplementary material for: Changes in mean serum lipids among adults in Germany: results from National Health Surveys 1997-99 and 2008-11
Source: BMC Public Health. 2016 Mar 8;16:240. doi: 10.1186/s12889-016-2826-2 (PMC4784325; doi:10.1186/s12889-016-2826-2)
Supplement: Additional file 2: Table S2. — Linear regression models for serum lipids with survey wave (DEGS1 vs. GNHIES98) as independent variable based on multiple imputed data. Table S3. Linear regression models for serum lipids with survey wave (DEGS1 vs. GNHIES98) as independent variable and additional explanatory variables. (DOCX 29 kb) [file 12889_2016_2826_MOESM2_ESM.docx]

Changes in mean serum lipids among adults in Germany: Results from National Health Surveys 1997-99 and 2008-11

Additional File 2

**Tab. S2** Linear regression models for serum lipids with survey wave (DEGS1 vs. GNHIES98) as independent variable based on multiple imputed data

|  | Men | | | | | Women | | | | |
| --- | --- | --- | --- | --- | --- | --- | --- | --- | --- | --- |
|  | Beta | 95% Confidence interval | | P | R²-range^d^ | Beta | 95% Confidence interval | | P | R²-range^d^ |
|  |  | Lower | Upper |  |  |  | Lower | Upper |  |  |
| **Total cholesterol (mmol/l)** | | | | | | | | | | |
| Model 1 | -0.858 | -0.939 | -0.777 | **<.001** | .1090 - .1117 | -0.792 | -0.869 | -0.716 | **<.001** | .0992 - .1002 |
| Model 2^a^ | -0.849 | -0.925 | -0.772 | **<.001** | .2494 - .2514 | -0.792 | -0.863 | -0.722 | **<.001** | .2990 - .3059 |
| Model 3^b^ | -0.800 | -0.878 | -0.722 | **<.001** | .2780 - .2811 | -0.794 | -0.870 | -0.719 | **<.001** | .3151 - .3226 |
| **Triglycerides (log-transformed; mmol/l)** | | | | | | | | | | |
| Model 1 | -0.178 | -0.212 | -0.144 | **<.001** | .0219 - .0233 | -0.106 | -0.140 | -0.073 | **<.001** | .0099 - .0109 |
| Model 2^a,c^ | -0.094 | -0.129 | -0.059 | **<.001** | .1256 - .1286 | -0.056 | -0.087 | -0.024 | **<.001** | .1593 - .1626 |
| Model 3^b,c^ | -0.087 | -0.123 | -0.052 | **<.001** | .2262 - .2315 | -0.058 | -0.089 | -0.03 | **<.001** | .2795 - .2849 |
| **High density lipoprotein cholesterol (mmol/l)** | | | | | | | | | | |
| Model 1 | -0.017 | -0.041 | 0.006 | .150 | .0005 - .0008 | -0.080 | -0.110 | -0.051 | **<.001** | .0085 - .0094 |
| Model 2^a^ | -0.017 | -0.040 | 0.006 | .150 | .0081 - .0092 | -0.080 | -0.109 | -0.051 | **<.001** | .0163 - .0169 |
| Model 3^b^ | -0.008 | -0.003 | 0.015 | .490 | .1312 - .1354 | -0.095 | -0.123 | -0.068 | **<.001** | .1540 - .1553 |

Differences between mean serum lipid levels were estimated with the t-test. P values< 0.05 were considered statistical significant (bold).

^a^ Adjusted for age

^b^ Adjusted for age, educational status, current smoking, coffee consumption, processed food consumption, wholegrain bread consumption, high alcohol consumption, sports activity, body mass index category, use of lipid-lowering medication, hormonal contraceptives, and postmenopausal hormone therapy

^c^ Additionally adjusted for fasting duration
^d^ R²-range: the range (min/max) of R² across the five imputed data sets

**Tab. S3** Linear regression models^a^ for serum lipids with survey wave (DEGS1 vs. GNHIES98) as independent variable and additional explanatory variables

|  | Men | | | | | Women | | | | |
| --- | --- | --- | --- | --- | --- | --- | --- | --- | --- | --- |
|  | Beta | 95% Confidence interval | | P | R² | Beta | 95% Confidence interval | | P | R² |
|  |  | Lower | Upper |  |  |  | Lower | Upper |  |  |
| **Total cholesterol (mmol/l)** | | | | | | | | | | |
| Model 3+HbA1c | -0.782 | -0.859 | -0.705 | **<.001** | .2857 | -0.789 | -0.869 | -0.710 | **<.001** | .3140 |
| Model 3+HT | -0.780 | -0.857 | -0.703 | **<.001** | .2834 | -0.786 | -0.863 | -0.709 | **<.001** | .3147 |
| Model 3+HT+DM | -0.774 | -0.851 | -0.697 | **<.001** | .2912 | -0.783 | -0.860 | -0.705 | **<.001** | .3165 |
| **Triglycerides (log-transformed; mmol/l)** | | | | | | | | | | |
| Model 3^b^+HbA1c | -0.085 | -0.120 | -0.051 | **<.001** | .2410 | -0.036 | -0.069 | -0.004 | **.029** | .3052 |
| Model 3^b^ +HT | -0.084 | -0.118 | -0.049 | **<.001** | .2356 | -0.050 | -0.084 | -0.017 | **.004** | .2845 |
| Model 3^b^ +HT+DM | -0.086 | -0.121 | -0.051 | **<.001** | .2361 | -0.060 | ´-0.093 | -0.028 | **004** | .2928 |
| **High density lipoprotein cholesterol (mmol/l)** | | | | | | | | | | |
| Model 3+HbA1c | -0.007 | -0.031 | 0.016 | .540 | .1356 | -0.110 | -0.139 | -0.083 | **<.001** | .1693 |
| Model 3+HT | -0.007 | -0.031 | 0.017 | .560 | .1329 | -0.098 | -0.126 | -0.070 | **<.001** | .1566 |
| Model 3+HT+DM | -0.006 | -0.030 | 0.018 | .630 | .1362 | -0.095 | -0.123 | -0.067 | **<.001** | .1635 |

HbA1c: glycated haemoglobin A1c (continuous), HT: hypertension (hypertensive blood pressure or intake of antihypertensive medication given that the person had known hypertension: yes, no), DM: known diabetes mellitus type II (yes, no)

^a^ Adjusted for age, educational status, current smoking, coffee consumption, processed food consumption, wholegrain bread consumption, high alcohol consumption, sports activity, body mass index category, use of lipid-lowering medication, hormonal contraceptives, and postmenopausal hormone therapy

^b^ Additionally adjusted for fasting duration
