# Supplementary material for: Changes in mean serum lipids among adults in Germany: results from National Health Surveys 1997-99 and 2008-11
Source: BMC Public Health. 2016 Mar 8;16:240. doi: 10.1186/s12889-016-2826-2 (PMC4784325; doi:10.1186/s12889-016-2826-2)
Supplement: Additional file 3: Table S4. — Linear regression models for total cholesterol. Table S5. Linear regression models for triglycerides. Table S6. Linear regression models for high density lipoprotein. (DOCX 47 kb) [file 12889_2016_2826_MOESM3_ESM.docx]

Changes in mean serum lipids among adults in Germany: Results from National Health Surveys 1997-99 and 2008-11

Additional File 3

**Tab. S4** Linear regression models for total cholesterol^a^

|  | | Men | | | | Women | | | |
| --- | --- | --- | --- | --- | --- | --- | --- | --- | --- |
|  |  | Beta | 95% Confidence interval | | P | Beta | 95% Confidence interval | | P |
|  |  |  | Lower | Upper |  |  | Lower | Upper |  |
| **Model 2** | | **R²=.2559** | | | | **R²=.2977** | | | |
| Survey wave (DEGS1 vs. GNHIES98) | | -0.828 | -0.904 | -0.753 | **<.001** | -0.782 | -0.855 | -0.708 | **<.001** |
| Age group | Spline-1 (20th percentile) | Reference |  |  |  | Reference |  |  |  |
|  | Spline-2 (40th percentile) | 0.071 | 0.064 | 0.078 | **<.001** | 0.019 | 0.014 | 0.024 | **<.001** |
|  | Spline-3 (60th percentile) | -0.003 | -0.004 | -0.003 | **<.001** | 0.003 | 0.003 | 0.004 | **<.001** |
|  | Spline-4 (80th percentile) | 0.005 | 0.003 | 0.006 | **<.001** | -0.008 | -0.009 | -0.006 | **<.001** |
| **Model 3** | | **R²=.2833** | | | | **R²=.3147** | | | |
| Survey wave (DEGS1 vs. GNHIES98) | | -0.779 | -0.856 | -0.702 | <.001 | -0.788 | -0.865 | -0.710 | <.001 |
| Age group | Spline-1 (20th percentile) | Reference |  |  |  | Reference |  |  |  |
|  | Spline-2 (40th percentile) | 0.064 | 0.057 | 0.070 | **<.001** | 0.018 | 0.013 | 0.024 | **<.001** |
|  | Spline-3 (60th percentile) | -0.003 | -0.003 | -0.002 | **<.001** | 0.004 | 0.003 | 0.005 | **<.001** |
|  | Spline-4 (80th percentile) | 0.004 | 0.002 | 0.005 | **<.001** | -0.008 | -0.010 | -0.007 | **<.001** |
| Educational status | Low | 0.006 | -0.099 | 0.110 | .910 | -0.005 | -0.110 | -0.099 | .290 |
|  | Middle | 0.036 | -0.068 | 0.141 | .500 | 0.051 | -0.044 | 0.147 | .290 |
|  | High | Reference |  |  |  | Reference |  |  |  |
| Current smoking | No | -0.138 | -0.225 | -0.051 | **.002** | -0.035 | -0.117 | 0.046 | .390 |
|  | Occasionally | -0.083 | -0.237 | 0.071 | .290 | -0.104 | -0.231 | 0.022 | .110 |
|  | Daily | Reference |  |  |  | Reference |  |  |  |
| Daily coffee consumption (yes vs. no) | | 0.063 | -0.012 | 0.139 | .099 | 0.069 | -0.009 | 0.147 | .083 |
| Daily processed foods consumption (yes vs. no) | | -0.013 | -0.097 | 0.071 | .770 | -0.028 | -0.090 | 0.035 | .380 |
| Wholegrain bread consumption | Never | -0.005 | -0.107 | 0.096 | .920 | -0.004 | -0.139 | 0.131 | .960 |
|  | Less than daily | 0.043 | -0.038 | 0.123 | .300 | 0.019 | -0.053 | 0.091 | .600 |
|  | Daily | Reference |  |  |  | Reference |  |  |  |
| High alcohol consumption (yes vs. no) | | 0.096 | 0.018 | 0.174 | **.016** | 0.033 | -0.060 | 0.125 | .490 |
| Sports activity | No | Reference |  |  |  | Reference |  |  |  |
|  | Up to 2h per week | -0.090 | -0.183 | 0.004 | .060 | -0.025 | -0.100 | 0.050 | .510 |
|  | Regularly, at least 2h per week | -0.092 | -0.201 | -0.016 | .095 | -0.075 | -0.167 | 0.016 | .100 |
| Body mass index category | Normal weight | -0.242 | -0.346 | -0.138 | **<.001** | -0.057 | -0.143 | 0.029 | .200 |
|  | Overweight | 0.068 | -0.026 | 0.162 | .150 | 0.102 | 0.007 | 0.197 | **.035** |
|  | Obese | Reference |  |  |  | Reference |  |  |  |
| Lipid-lowering medication (yes vs. no) | | -0.536 | -0.684 | 0.388 | **<.001** | -0.504 | -0.656 | 0.351 | **<.001** |
| Hormonal contraceptives (yes vs. no) | | - | - | - | - | 0.164 | 0.085 | 0.243 | **<.001** |
| Postmenopausal hormone therapy (yes vs. no) | | - | - | - | - | -0.169 | -0.275 | -0.064 | **.002** |

Differences between mean serum lipid levels were estimated with the t-test. P values< 0.05 were considered statistical significant (bold).

^a^ All models are weighted population estimates: standardized to population structure as of 31^st^ December 2010

**Tab. S5** Linear regression models for triglycerides^a^

|  | | Men | | | | Women | | | |
| --- | --- | --- | --- | --- | --- | --- | --- | --- | --- |
|  |  | Beta | 95% Confidence interval | | P | Beta | 95% Confidence interval | | P |
|  |  |  | Lower | Upper |  |  | Lower | Upper |  |
| **Model 2** | | **R²=.1298** | | | | **R²=.1584** | | | |
| Survey wave (DEGS1 vs. GNHIES98) | | -0.088 | -0.122 | -0.054 | **<.001** | -0.050 | -0.084 | -0.017 | **.004** |
| Age group | Spline-1 (20th percentile) | Reference |  |  |  | Reference |  |  |  |
|  | Spline-2 (40th percentile) | 0.021 | 0.018 | 0.025 | **<.001** | 0.002 | -0.001 | 0.005 | .220 |
|  | Spline-3 (60th percentile) | -0.001 | -0.002 | -0.001 | **<.001** | 0.001 | 0.001 | 0.002 | **<.001** |
|  | Spline-4 (80th percentile) | 0.001 | 0.001 | 0.002 | **<.001** | -0.002 | -0.003 | -0.002 | **<.001** |
| Fasting duration | < 4 h | 0.371 | 0.321 | 0.421 | **<.001** | 0.280 | 0.243 | 0.315 | **<.001** |
|  | 4 – 8 h | 0.178 | 0.139 | 0.216 | **<.001** | 0.101 | 0.072 | 0.130 | **<.001** |
|  | > 8 h | Reference |  |  |  | Reference |  |  |  |
| **Model 3** | | **R²=.2315** | | | | **R²=.2817** | | | |
| Survey wave (DEGS1 vs. GNHIES98) | | -0.081 | -0.115 | -0.047 | **<.001** | -0.050 | -0.084 | -0.017 | **.003** |
| Age group | Spline-1 (20th percentile) | Reference |  |  |  | Reference |  |  |  |
|  | Spline-2 (40th percentile) | 0.015 | 0.012 | 0.018 | **<.001** | 0.002 | -0.001 | 0.005 | .130 |
|  | Spline-3 (60th percentile) | -0.001 | -0.001 | 0.000 | **<.001** | 0.001 | 0.001 | 0.002 | **<.001** |
|  | Spline-4 (80th percentile) | 0.001 | 0.001 | 0.002 | **.001** | -0.003 | -0.003 | -0.001 | **<.001** |
| Fasting duration | < 4 h | 0.358 | 0.313 | 0.404 | **<.001** | 0.258 | 0.226 | 0.290 | **<.001** |
|  | 4 – 8 h | 0.186 | 0.150 | 0.222 | **<.001** | 0.099 | 0.072 | 0.127 | **<.001** |
|  | > 8 h | Reference |  |  |  | Reference |  |  |  |
| Educational status | Low | 0.022 | -0.034 | 0.077 | .440 | 0.014 | -0.026 | 0.055 | .490 |
|  | Middle | 0.002 | -0.045 | 0.049 | .930 | -0.009 | -0.049 | 0.031 | .650 |
|  | High | Reference |  |  |  | Reference |  |  |  |
| Current smoking | No | -0.114 | -0.152 | -0.076 | **<.001** | -0.152 | -0.192 | -0.113 | **<.001** |
|  | Occasionally | 0.005 | -0.072 | 0.081 | .900 | -0.101 | -0.158 | -0.043 | **.001** |
|  | Daily | Reference |  |  |  | Reference |  |  |  |
| Daily coffee consumption (yes vs. no) | | -0.065 | -0.104 | -0.027 | **.001** | -0.030 | -0.062 | 0.001 | .057 |
| Daily processed foods consumption (yes vs. no) | | -0.031 | -0.061 | 0.001 | **.045** | -0.033 | -0.057 | -0.009 | **.008** |
| Wholegrain bread consumption | Never | 0.036 | -0.014 | 0.085 | .160 | 0.030 | -0.019 | 0.078 | .230 |
|  | Less than daily | 0.041 | 0.005 | 0.077 | **.027** | 0.022 | -0.005 | 0.048 | .110 |
|  | Daily | Reference |  |  |  | Reference |  |  |  |
| High alcohol consumption (yes vs. no) | | 0.018 | -0.023 | 0.058 | .390 | -0.004 | -0.047 | 0.038 | .840 |
| Sports activity | No | Reference |  |  |  | Reference |  |  |  |
|  | Up to 2h per week | -0.059 | -0.100 | -0.018 | **.005** | -0.036 | -0.066 | -0.006 | **.020** |
|  | Regularly, at least 2h per week | -0.072 | -0.109 | -0.035 | **<.001** | -0.058 | -0.097 | -0.020 | **.003** |
| Body mass index category | Normal weight | -0.474 | -0.529 | -0.420 | **<.001** | -0.390 | -0.429 | -0.351 | **<.001** |
|  | Overweight | -0.210 | -0.251 | -0.168 | **<.001** | -0.189 | -0.229 | -0.149 | **<.001** |
|  | Obese | Reference |  |  |  | Reference |  |  |  |
| Lipid-lowering medication (yes vs. no) | | 0.165 | 0.109 | 0.222 | **<.001** | 0.064 | 0.002 | 0.126 | **.044** |
| Hormonal contraceptives (yes vs. no) | | - | - | - | - | 0.212 | 0.173 | 0.251 | **<.001** |
| Postmenopausal hormone therapy (yes vs. no) | | - | - | - | - | -0.028 | -0.088 | 0.031 | .350 |

Differences between mean serum lipid levels were estimated with the t-test. P values< 0.05 were considered statistical significant (bold).

^a^ All models are weighted population estimates: standardized to population structure as of 31^st^ December 2010

**Tab S6** Linear regression models for high density lipoprotein^a^

|  | | Men | | | | Women | | | |
| --- | --- | --- | --- | --- | --- | --- | --- | --- | --- |
|  |  | Beta | 95% Confidence interval | | P | Beta | 95% Confidence interval | | P |
|  |  |  | Lower | Upper |  |  | Lower | Upper |  |
| **Model 2** | | **R²=.0089** | | | | **R²=.0168** | | | |
| Survey wave (DEGS1 vs. GNHIES98) | | -0.016 | -0.039 | 0.007 | .170 | -0.081 | -0.111 | -0.051 | **<.001** |
| Age group | Spline-1 (20th percentile) | Reference |  |  |  | Reference |  |  |  |
|  | Spline-2 (40th percentile) | 0.001 | -0.001 | 0.003 | .190 | 0.003 | 0.001 | 0.006 | **.001** |
|  | Spline-3 (60th percentile) | 0.000 | 0.000 | 0.000 | .094 | 0.000 | 0.000 | 0.000 | .740 |
|  | Spline-4 (80th percentile) | 0.000 | -0.001 | 0.000 | .056 | 0.000 | -0.001 | 0.000 | .260 |
| **Model 3** | | **R²=.1330** | | | | **R²=.1571** | | | |
| Survey wave (DEGS1 vs. GNHIES98) | | -0.007 | -0.031 | 0.017 | .560 | -0.099 | -0.127 | -0.071 | **<.001** |
| Age group | Spline-1 (20th percentile) | Reference |  |  |  | Reference |  |  |  |
|  | Spline-2 (40th percentile) | 0.004 | 0.002 | 0.006 | **<.001** | 0.005 | 0.003 | 0.008 | **<.001** |
|  | Spline-3 (60th percentile) | 0.000 | 0.000 | 0.000 | .490 | 0.000 | 0.000 | 0.000 | .500 |
|  | Spline-4 (80th percentile) | 0.000 | -0.001 | 0.000 | .190 | 0.000 | -0.001 | 0.000 | .180 |
| Educational status | Low | 0.012 | -0.018 | 0.042 | .430 | -0.060 | -0.100 | -0.019 | **.004** |
|  | Middle | 0.024 | -0.006 | 0.054 | .110 | -0.024 | -0.059 | 0.011 | .170 |
|  | High | Reference |  |  |  | Reference |  |  |  |
| Current smoking | No | 0.094 | 0.071 | 0.117 | **<.001** | 0.142 | 0.113 | 0.171 | **<.001** |
|  | Occasionally | 0.040 | -0.004 | 0.083 | .070 | 0.130 | 0.086 | 0.173 | **<.001** |
|  | Daily | Reference |  |  |  | Reference |  |  |  |
| Daily coffee consumption (yes vs. no) | | 0.033 | 0.006 | 0.059 | **.017** | 0.062 | 0.031 | 0.092 | **<.001** |
| Daily processed foods consumption (yes vs. no) | | -0.001 | -0.022 | 0.020 | .910 | -0.023 | -0.046 | -0.001 | **.049** |
| Wholegrain bread consumption | Never | -0.030 | -0.059 | -0.001 | **.042** | -0.024 | -0.076 | 0.028 | .370 |
|  | Less than daily | -0.004 | -0.026 | 0.019 | .750 | -0.013 | -0.034 | 0.009 | .250 |
|  | Daily | Reference |  |  |  | Reference |  |  |  |
| High alcohol consumption (yes vs. no) | | 0.163 | 0.136 | 0.190 | **<.001** | 0.162 | 0.126 | 0.198 | **<.001** |
| Sports activity | No | Reference |  |  |  | Reference |  |  |  |
|  | Up to 2h per week | 0.011 | -0.012 | 0.033 | .350 | 0.014 | -0.013 | 0.040 | .310 |
|  | Regularly, at least 2h per week | 0.028 | 0.003 | 0.052 | **.026** | 0.028 | -0.004 | 0.061 | .084 |
| Body mass index category | Normal weight | 0.260 | 0.228 | 0.292 | **<.001** | 0.307 | 0.274 | 0.341 | **<.001** |
|  | Overweight | 0.118 | 0.095 | 0.141 | **<.001** | 0.163 | 0.129 | 0.197 | **<.001** |
|  | Obese | Reference |  |  |  | Reference |  |  |  |
| Lipid-lowering medication (yes vs. no) | | -0.071 | -0.104 | -0.038 | **<.001** | -0.025 | -0.070 | 0.020 | .270 |
| Hormonal contraceptives (yes vs. no) | | - | - | - | - | 0.080 | 0.050 | 0.110 | **<.001** |
| Postmenopausal hormone therapy (yes vs. no) | | - | - | - | - | 0.037 | -0.014 | 0.088 | .150 |

Differences between mean serum lipid levels were estimated with the t-test. P values< 0.05 were considered statistical significant (bold).

^a^ All models are weighted population estimates: standardized to population structure as of 31^st^ December 2010
